# A Mechanistic Exploratory Study on the Therapeutic Efficacy of Astragaloside IV Against Diabetic Retinopathy Revealed by Network Pharmacology

**DOI:** 10.3389/fphar.2022.903485

**Published:** 2022-06-22

**Authors:** Zhi-Hao Zhao, Min Xu, Cong Fu, Ying Huang, Ting-Hua Wang, Zhong-Fu Zuo, Xue-Zheng Liu

**Affiliations:** ^1^ Department of Anatomy, Histology and Embryology, Jinzhou Medical University, Jinzhou, China; ^2^ Liaoning Key Laboratory of Diabetic Cognitive and Perceptive Dysfunction, Jinzhou Medical University, Jinzhou, China; ^3^ Institute of Neuroscience, Laboratory Animal Department, Kunming Medical University, Kunming, China

**Keywords:** diabetic retinopathy, astragaloside IV, bioinformatics analysis, multiple targets, PI3K-AKT signaling pathway

## Abstract

**Purpose:** Diabetic retinopathy (DR) is a serious complication of diabetes mellitus, which nearly happens to all the diabetic sufferers. This study aims to identify the preliminary molecular regulation involved in the therapeutic efficacy of astragaloside IV (AS- IV) for DR.

**Methods:** Diabetic rat models were established and treated with AS-IV. Optical coherence tomography (OCT) and Hematoxylin-eosin (HE) staining was employed to demonstrate the histopathological changes. The main targets of AS-IV were identified by searching from public databases of traditional Chinese medicine (GeneCards, PharmMapper and Swiss Target Prediction). Besides, disease targets of DR were also obtained by integrated data from GEO datasets and predicted from public databases. Protein-protein interaction (PPI) network was constructed by Cytoscape with overlapping genes and 10 core targets were selected, on which Gene Ontology (GO) along with Kyoto Encyclopedia of Genes and Genomes (KEGG) enrichment analysis were conducted. The interaction between AS-IV and these crucial genes were analyzed using molecular docking. RT-qPCR and western blot were used to verify the expression variation of core targets.

**Results:** OCT imaging and HE staining demonstrated that AS-IV administration significantly increased retinal thickness in diabetic rats, obviously alleviating DR induced histopathological changes as well as elevated blood glucose levels. 107 common targets of AS-IV and DR were determined after intersection. PPI network analysis filtered 10 hub genes potentially targeted by AS-IV, including VEGFA, CASP3, HIF1α, STAT3, CTNNB1, SRC, AKT1, EGFR, IL1β and IL6. Enrichment analysis indicated that these genes were mainly enriched in biological processes like T cell activation, epithelial cell proliferation and protein kinase B signaling, and involved in oxidative stress, apoptosis and inflammation-related pathways. The molecular docking prediction suggested that AS-IV exhibited stable binding to these core targets. In addition, mRNA levels of core targets in diabetic rats were differentially expressed before and after AS-IV treatment. Western blot further revealed that AS-IV treatment elevated DR-depressed protein levels of PI3K and AKT.

**Conclusion:** Our study elucidated the effect of AS-IV in attenuating retinopathy induced by diabetes in rats and preliminarily unveiled the therapeutic efficacy of AS-IV in the treatment of DR might be attributed to activation of PI3K-AKT signaling pathway.

## Introduction

Diabetic retinopathy (DR) represents as the most common and serious ocular complication of diabetes, with approximately 103 million DR patients worldwide as of 2020 and an estimated number of 160 million DR patients in 2045 (Teo, et al., 2021). DR causes irreversible visual impairment of patients, and presents with clinical manifestations such as visual abnormalities, blurred vision, and even blindness, which is the leading cause of blindness in adults in countries all over the world. In addition to its impact on vision, the presence of DR implies an increased risk of life-threatening systemic vascular complications (Cheung, et al., 2010). Also, DR leads to a poor quality of life and also increases the risk of other diabetic comorbidities and mortality, imposing a serious burden on society (Kramer, et al., 2011). Presently, the main treatments for DR include retinal laser photocoagulation, drug therapy, hormone therapy, and surgery. However, these treatments may result in undesirable effects such as increased angiogenesis, elevated intraocular pressure and retinal hemorrhage (Stitt, et al., 2016; Whitcup, et al., 2018). Studies have shown that age is a critical factor influencing DR, with a higher number of DR patients in older populations (Youngblood, et al., 2019). As global aging continues to develop, it is of great significance to actively explore the pathogenesis of DR and explore effective therapeutic drugs.

Traditional Chinese medicine (TCM) has been used in the prevention and treatment of chronic diseases for nearly 2,000 years and has made indelible contributions. TCM believes that the pathogenesis of DR can be derived from deficiency of both *qi* and *yin*. On the basis, people may develop the types of *yin* deficiency and fire, *yin* deficiency and stomach heat, *yin* deficiency in lung and kidney, *yin* deficiency in liver and kidney, *yin* and *yang* loss, etc. (Ai, et al., 2020). Although the pathogenesis and symptoms of DR varies, the key to the pathogenesis of DR lies in the stagnation of the eyelid channels and nutritional deficiencies of the eyes. Astragalus is the holy medicine for supplementing *qi*, which is listed as top grade by *Shennong Materia Medica*, and has the function of tonifying *qi* and dredging collaterals. Previous practice shows that “*Huangqi Decoction*” and “*Huangqi injection*” exert obvious effects in the prevention and treatment of diabetes induced neuropathy (Chang, et al., 2017; Zhang, et al., 2019). Astragaloside IV (AS-IV) is the main pharmacological active component of *Astragalus membranaceus*, which has anti-inflammatory, hypotensive, anti-diabetes and myocardial protective properties (Gui, et al., 2013; He, et al., 2013; Zhang, et al., 2020). In addition, AS-IV significantly suppresses iron death in retinal pigment epithelial cells (Tang, et al., 2022), inhibits the formation of late glycation end products (Motomura, et al., 2009), and also reduces apoptotic retinal ganglion cell by reducing phosphorylation of ERK1/2 and inhibiting activation of NF-κB and various cytokines (Hua, et al., 2019). Despite great potentials AS-IV exhibits in the treatment of DR, the protective mechanism of AS-IV involved in DR remains ambiguous.

**FIGURE 1:**
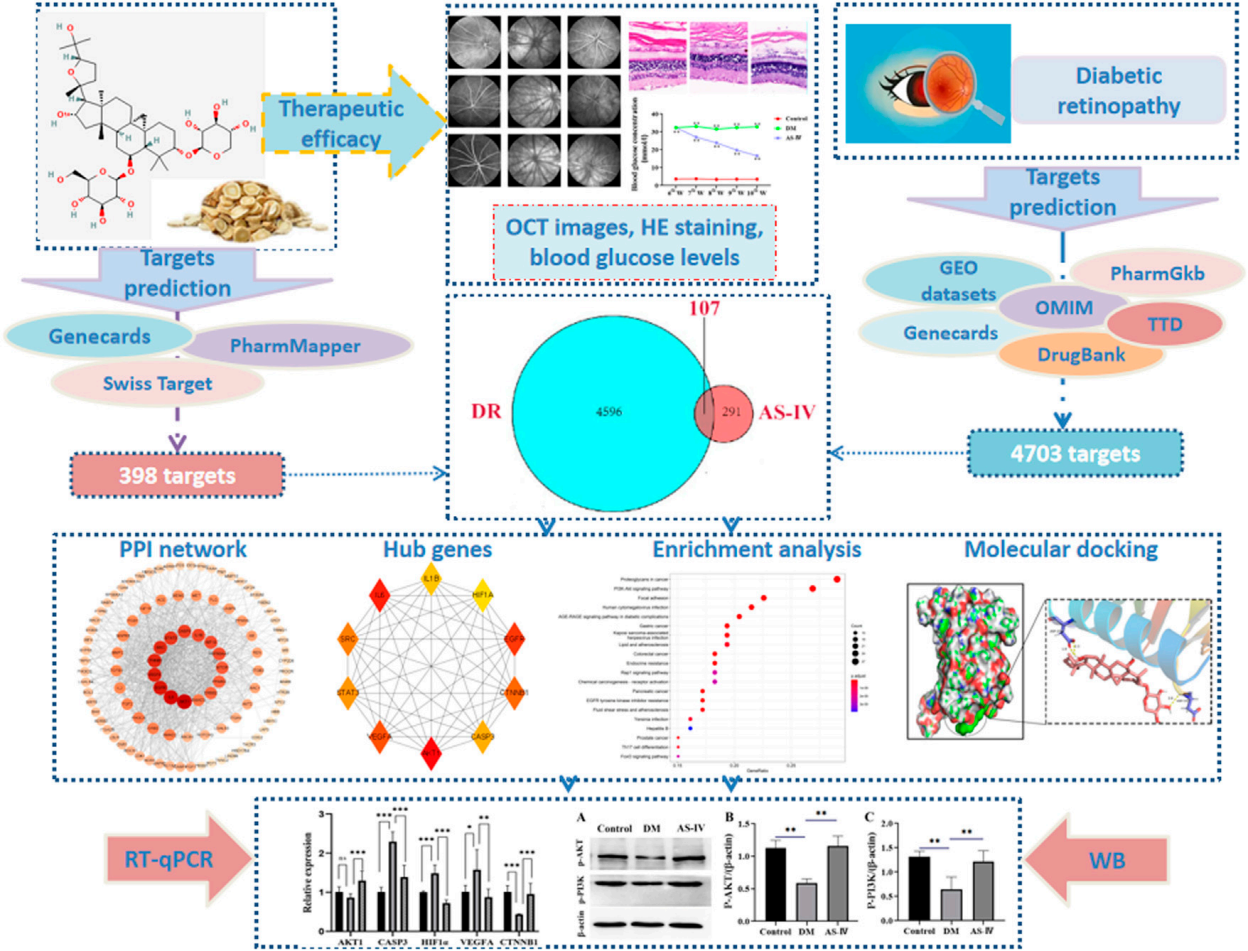
Flow chart of study design. DR, diabetic retinopathy; AS-IV, astragaloside IV; RT-qPCR, real time quantitative polymerase chains reaction; WB, western blot.

With the rapid development of bioinformatics, system biology and comprehensive pharmacology, network pharmacology based on the concept of “drug-disease target-pathway” can explore the complex action mechanism of drugs on human body (Hopkins, 2008). Meanwhile, network pharmacology, based on system bioinformatics, can analyze the complex target and target signal network, which helps evaluate the feasibility and applicability of TCM in treating complex diseases. In this study, to investigate the molecular mechanism underlying the therapeutic effect of AS-IV in DR, multiple databases were used to screen potential drug targets of AS-IV and disease targets of DR, so as to obtain the AS-IV-DR composite targets. Protein-protein interaction (PPI) network is constructed to analyze connection among these targets and identify the hub genes in accordance with the topological interaction between these targets. Afterwards, enrichment analysis of Kyoto Encyclopedia of Genes and Genomes (KEGG) and Gene Ontology (GO) were employed to elucidate the biological functions involved by hub genes. Finally, molecular docking techniques along with *in vivo* experiments were applied to verify the reliability of these crucial targets (Figure 1).

## Materials and Methods

### Animal Grouping and Treatment

Male Sprague Dawley (SD) rats (180–220 g) were purchased from Experimental Animal Center of Kunming Medical University. The rats were kept in a controlled room at 22 ± 2.0°C and 50% ± 5% humidity, with light exposure for about 12 h a day and free access to food and water. One week after adaptive feeding, rats were injected intraperitoneally with streptozotocin (60 mg/kg, Merck reagent, lot V900890) dissolved in a freshly prepared sodiumcitrate buffer (50 mM, pH4.5) so as to induce diabetes mellitus (Du et al., 2017). Age-matched control rats received the equivalent volume of vehicles. Seventy-2 h after injection, blood samples were collected from the tail of the rats (fasted for 12 h) for the measurement of glucose levels. Rats with fasting blood glucose levels exceeding 16.7 mmol/L for three consecutive days (Sun, et al., 2020) were considered as diabetic rats and were used as animal models for subsequent experiments. AS-IV reduced the blood glucose levels of diabetes rats in a dose-dependent manner (Du et al., 2017), higher dose treatment exhibits better hypoglycemic effects. SD rats were divided into three groups (n = 8/group): 1) control group: normal rats were treated with normal saline (10 ml/kg); 2) diabetic group: diabetic rats received normal saline treatment (10 ml/kg); 3) AS-IV group: diabetic rats were intragastrically treated with AS-IV (100 mg/kg). Six weeks after diabetic insult, rats received AS-IV treatment consecutively for 28 days. The fasting blood glucose levels of rats were measured once a week.

### Optical Coherence Tomography

Rats were anesthetized with intraperitoneal injection of 3% phenobarbital (50 mg/kg). Compound topicamide eye drops were applied externally to dilate the pupil, and flufloxacin protected the cornea during and after surgery. Rats in the control group, diabetic group, and AS-IV treatment group were recorded using the Micron IV Retinal imaging camera system (Phoenix Research Laboratory, Pleasanton, CA). Finally, the retinal thickness of OCT images was measured by ImageJ software.

### Tissue Harvest

The blood glucose of rats was decreased on the 7th, 14th, 21st and 28th day after administration and the retinal thickness was increased on the 28th day after administration (Liu et al., 2010). Accordingly, rats in the AS-IV group were treated with 100 mg/kg AS-IV for 28 days, and then were killed with the eyeballs harvested. The collected eyeball was placed in a Petri dish with normal saline on ice for retinal separation. The eyeball wall was incised with a scalpel at the equivalent of the ciliary flattening, and ophthalmic scissors were used to expand the incision until the anterior segment was completely removed. Then, the posterior segment wall was reversed for vitreous tilting. After a fraction of retina was separated, ophthalmic forceps was applied to separate the retina to the optic papilla, and finally the root was cut out with ophthalmic scissors. The removed retina was placed into a new EP tube.

### AS-IV Related Target Prediction

The two-dimensional molecular structure of AS-IV was obtained from PubChem (https://pubchem.ncbi.nlm.nih.gov/) (Liu et al., 2010), the largest free database of chemical information in the world. Firstly, the targets of AS-IV were predicted in Genecards (https://www.genecards.org/). Meanwhile, the two-dimensional molecules of AS-IV were uploaded into Swiss Target Prediction database (http://www.swisstargetprediction.ch/) as well as into PharmMapper database (http://lilab.ecust.edu.cn/pharmMapper/) respectively for target prediction (Liu et al., 2010), with species defined as “*Homo sapiens*”. Additionally, Uniprot protein database (https://www.uniprot.org/) (UniProt, 2021) was used to retrieve the predicted targets, and the protein targets and genes of AS-IV were standardized and integrated.

### Integration of Affymetrix Microarray Data

We searched the raw data of DR from the Comprehensive Gene Expression Database (http://www.ncbi.nlm.nih.gov/geo). The GSE12610 dataset contained 5 RNA samples extracted from the retinas of musculus including 3 diabetic samples (streptozotocin induced diabetes at 5 weeks) and 2 normal samples. In GSE28831 dataset, the retinas of Evans rats were extracted and microarray experiments were performed on days 7, 28, and 84 after STZ-induced diabetes. GPL20710 [MoGene-2_0-ST] Affymetrix Mouse Gene 2.0 ST array (mogene20st_Mm_ENTREZG_19) and GPL7294 (Agilent-014879 whole rat genome microarray 4x44KG4131F) were used to analyze datasets GSE12610 and GSE28831. Both datasets identified genes and pathways involved in DR formation. The R programming language was used for statistical analysis and graphing, and Pearson correlation analysis was performed to verify the repeatability of the data within each group. Heat maps were generated to visualize correlations among all samples from the same dataset.

### Identification of Differentially Expressed Genes and Construction of DR Targets

Microarray data linear module Bioconductor (Robinson, et al., 2010) identified DEGs by comparing their expression values in the retinas of normal and diabetic mice, with adjusted *p* < 0.05 and log2 fold change >1 or < −1 as selection criteria. The heat map of the DEGs was plotted using heat map package in R4.1.1. In addition, DR-related target genes were searched in five public databases: Genecards database (https://www.genecards.org/) (Fishilevich, et al., 2017), OMIM database (https://omim.org/) (Amberger, et al., 2015), PharmGkb database (https://www.pharmgkb.org/) (Barbarino, et al., 2018), TTD database (http://db.idrblab.net/ttd/) (Chen, et al., 2002) and DrugBank database, with “diabetic retinopathy” as the key word. Combined with results from Uniprot database (https://www.uniprot.org/) (UniProt, 2021) and GEO datasets, a disease target database of DR was established after elimination of redundant disease targets.

### Construction of Protein-Protein Interaction Networks and Enrichment Analysis on Biological Function

In order to clarify the interaction between AS-IV related targets and DR targets, we screened the overlapping genes between AS-IV related targets and DR related targets, which were then imported into the STRING database (https://string-db.org/) (von Mering, et al., 2005) for interaction analysis. The analyzed results were saved as TSV files and imported into Cytoscape 3.8.2 for network generation. The plug-in “cytoHubba” was applied for network topology analysis in Cytoscape. In addition, these plug-ins of R programming “clusterProfiler”, “org.hs.eg.db”, “EnrichPlot” and “GGploT2” were used to carry out the GO and KEGG pathways analysis of those overlapping targets. GO analysis annotates genes and gene products based on molecular function (MF), biological process (BP), and cellular composition (CC). KEGG is a useful resource for systematic analysis of gene function and related high-level genomic function information.

### Molecular Docking

Molecular docking is a computer structure-based method widely used in drug discovery, which can identify new compounds of therapeutic significance, predict ligand target interactions at molecular level, and depict the structure-activity relationship (SAR) in absence of chemical structure of other target regulators in advance (Pinzi and Rastelli, 2019; Wang et al., 2020). The high-resolution crystal structure of active components and their corresponding bioactive ligands can be obtained from the protein database (PDB), a single global repository of the 3D structure of biological macromolecules and their complexes determined experimentally (Burley, et al., 2017). We used PyMOL software to dehydrate the target protein structure and separate ligands and receptors, the target protein taken as the receptor and AS-Ⅳ as the ligand. The active site of molecular docking was determined according to the coordinates of the ligand in the target protein complex, and the Gridbox coordinates and size were set according to the active pocket of the target protein. Subsequently, molecular docking was performed in Autodock vina. Finally, the interaction between protein and ligand was studied by means of binding energy scores. The obtained docking protein structures were further optimized using PyMOL software.

### Hematoxylin-Eosin Staining

The collected eyeballs were fixed with 4% paraformaldehyde for 1 h, and then dehydrated with sucrose solution. Subsequently, the eyeballs were embedded in the optimal cutting temperature compound medium (Sakura tissue TEK, West Chester, PA) and then stored at −80°C fridge overnight. Retinal frozen sections were prepared at thickness of 10 μm using Leica microtome (CM1860, Leica Microsystems, Germany). These sections were subsequently treated with hematoxylin solution (Servicebio, G1005) for 1 min, after washes under running water, and they were continuingly stained by eosin solution (Servicebio, G1005) for 2–3 min. After sections dehydrated and transparentized, the fields near the optic nerve (200 μm) were captured for analysis.

### RT-qPCR

Total RNA was extracted from the harvested retinal tissues using the Trizol kit (Biosharp), and cDNA was reversely transcribed using reverse transcriptase kit (Promega). Later, amplification reaction was followed with 20 μL reaction system composed of 10 μL SYBR-Green qPCR Master Mix, 1 μL cDNA Template, 7.8 μL DEPC water and 0.6 μL each of forward and reverse primers. Primers sequences are shown in Table 1. The thermal reaction cycle of PCR reaction was: one cycle of initial denaturation at 95°C for 2 min; 40 cycles of denaturation at 95°C for 15 s, annealing at 59°C for 20 s, and elongation at 60°C for 40 s. GAPDH was used as an internal reference gene, and the relative expression were calculated using the 2^−ΔΔCt^ method.

**TABLE 1:** Primer sequences of detected genes.

Gene name	Forward	Reverse
VEGFA	TTA​CTG​CTG​TAC​CTC​CAC​CAT	CGT​CCA​TGA​ACT​TCA​CCA​CTT
CASP3	TGC​GGT​ATT​GAG​ACA​GAC​AGT	GCG​GTA​GAG​TAA​GCA​TAC​AGG​A
HIF1A	GCC​ACC​ACC​ACT​GAT​GAA​TC	CCA​CTG​TAT​GCT​GAT​GCC​TTA​G
STAT3	TGT​CAG​ATG​CCT​AAT​GCT​TGG	TGG​TGG​TGG​ACG​AGA​ACT​G
CTNNB1	TGG​CAG​CAG​CAA​TCT​TAC​CT	GTG​TCC​ACA​TCT​TCT​TCC​TCA​G
SRC	TGTCACCGTCTCACTACC	TTCTCGCCAACCAGGATA
AKT1	CAG​GAG​GAG​GAG​ACG​ATG​GA	ATG​GTC​ACA​CGG​TGC​TTG​G
EGFR	GAG​GTG​GCT​GGC​TAT​GTT​CT	CGG​CTA​AGG​CGT​AGG​TGT​T
IL1β	CTC​ATT​GTG​GCT​GTG​GAG​AAG	ACA​CTA​GCA​GGT​CGT​CAT​CAT
IL6	CCA​GCC​AGT​TGC​CTT​CTT​G	AAT​TAA​GCC​TCC​GAC​TTG​TGA​A

### Western Blot

Retinal tissues were lysed with 100 μL of RIPA tissue lysates, and the supernatant was discarded after centrifugation (15,000 rpm) at 4°C for 15 min. Then, the protein concentration and purification were determined by BCA method. The protein extracts were separated with agarose gel electrophoresis, and then transferred to polyvinylidene fluoride (PVDF) membranes. The PVDF membranes were placed in solution containing 5% fat-free milk powder and blocked with gentle shaking at room temperature for 1 h. Afterwards, these membranes were incubated with primary polyclonal antibodies against p-PI3K(1:1,000, rabbit, Bioss, bs-20611R) and p-AKT (1:1,000, rabbit, Bioss, bs-2720R) and β-actin monoclonal antibodies (1:5,000, mouse, Proteintech, 66009-1-Ig) overnight at 4°C. After washing three times, secondary antibodies (IgG-HRP, 1:5,000, goat anti-rabbit, Abbkine, A21031; IgG-HRP, 1:5,000, goat anti-mouse, Abbkine, A21010) were added to incubate for 1 h. After washing, the protein bands were developed with ECL chemiluminescence substrate kit (BL520B, Biosharp). β-actin was used as a loading control. Finally, densitometry analysis was performed using ImageJ software, and the relative expression levels were calculated by using the gray value of the target strip than the gray value of β -actin.

### Statistical Analysis

All data in this study were processed by SPSS21.0 statistical software (IBM) or R programming. Measurement data were expressed by mean ± standard deviation $\left(\bar{x}\pm \mathrm{SD}\right)$ , and comparison among groups was performed by one way ANOVA. *p* < 0.05 is considered statistically significant.

## Results

### The Protective Effect of AS-IV on DR Retina

**FIGURE 2:**
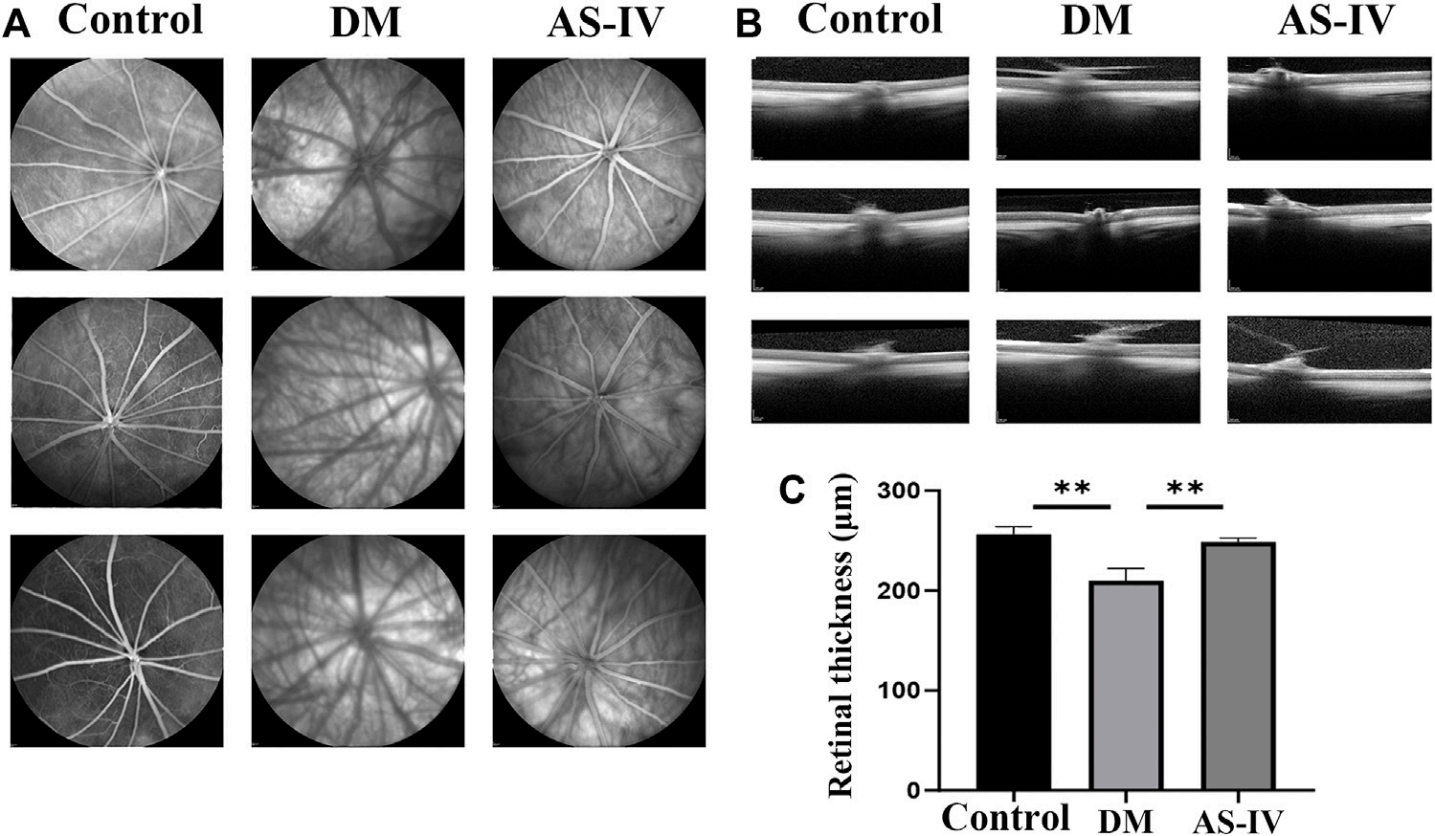
The effect of AS-IV treatment on retinal vessels in diabetic rats. **(A)** Retinal vascular images of rats in control group, diabetic group and treatment groups. **(B)** The images of retina in control group, diabetic group and treatment groups. **(C)** Quantification of retinal thickness among three groups. DM, diabetes mellitus; AS-IV, astragaloside IV. **p < 0.01.

We firstly verified the effects of AS-IV treatment on retinal condition after DR by employing OCT on rats in the control group, the diabetic group and the treatment group. As shown in the representative images, detailed retinal layer and fundus photographs were observed in all groups, and no significant malformations were shown in any diabetic rats (Figure 2). However, the total retinal thickness of the diabetic model rats was significantly reduced compared with that of the control group, and the fundus vascular images of the diabetic model rats were blurrier as a result of vitreous opacity and corneal whiteness of the diabetic rats (Figure 2A). Meanwhile, a large number of new vessels appeared disorderly in the fundus of the diabetic rats, which were prominently decreased in the diabetic rats treated with AS-IV (Figure 2A). Additionally, the thinner retinas with different degrees of cavities and cell loss in diabetic rats became thicker after AS-IV treatment than those in the diabetic group (Figures 2B,C, *p* < 0.01).

### AS-IV Alleviated DR Induced Histopathological Changes in Rats

**FIGURE 3:**
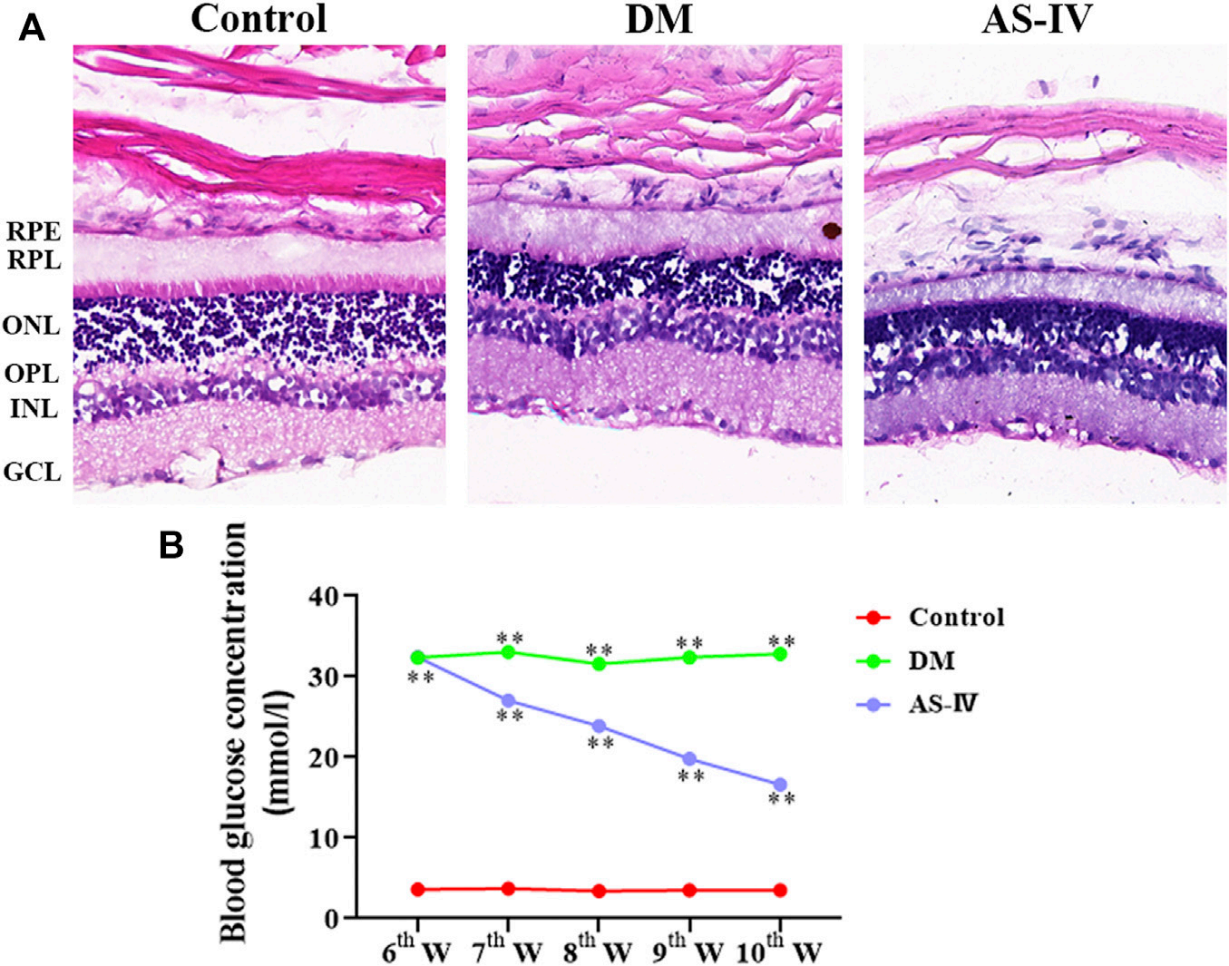
Effects of AS-IV on histopathological changes and blood glucose levels of rats after DR. **(A)** HE staining images of retinal tissues in rats among control, DM and AS-IV groups. Scale bar = 200 μm. **(B)** Blood glucose levels variation of rats in control, DM and AS-IV treatment groups. RPL, retinal photoreceptor layer; RPE, retinal pigment epithelium; ONL, outer nuclear layer; GCL, ganglion cell layer; INL, inner nuclear layer; OPL, outer plexiform layer; DM, diabetes mellitus; AS-IV, astragaloside IV; w, weeks. **p < 0.01.

The morphological and histopathological changes of rat retina after DR were observed by HE staining. The retinal surface in the control group was smooth. Each layer presents a clear and complete structure, and the cells in each layer are arranged orderly, evenly and densely (Figure 3A). However in DM group, the cells in each layer of retina were arranged disorderly, and ganglion cells were denatured or necrotic. In addition, the thickened optic nerve fiber layer s, nerve fiber edema, inflammatory cell infiltration and vacuole formation were observed in the inner core and outer plexus layer (Figure 3A). These alterations corresponded to the basic pathological characteristics of DR. After treatment with AS-IV, the pathological changes induced by DR were largely alleviated (Figure 3A). The efficacy of AS-IV in DR rats was also demonstrated by long-term blood glucose levels monitoring. As revealed, the fasting blood glucose levels of rats in DM group and treatment group were much higher (>16.7 mmol/L) than that of control group at the 6^th^ week prior to AS-IV treatment (Figure 3B, *p* < 0.01). There was no significant change in blood glucose of rats in control group as time prolonged. After AS-IV treatment, blood glucose levels decreased a lot within the later 4 weeks, which were significantly lower than that of DM group (Figure 3B, *p* < 0.01).

### Targets Prediction of AS-IV and DR

**FIGURE 4:**
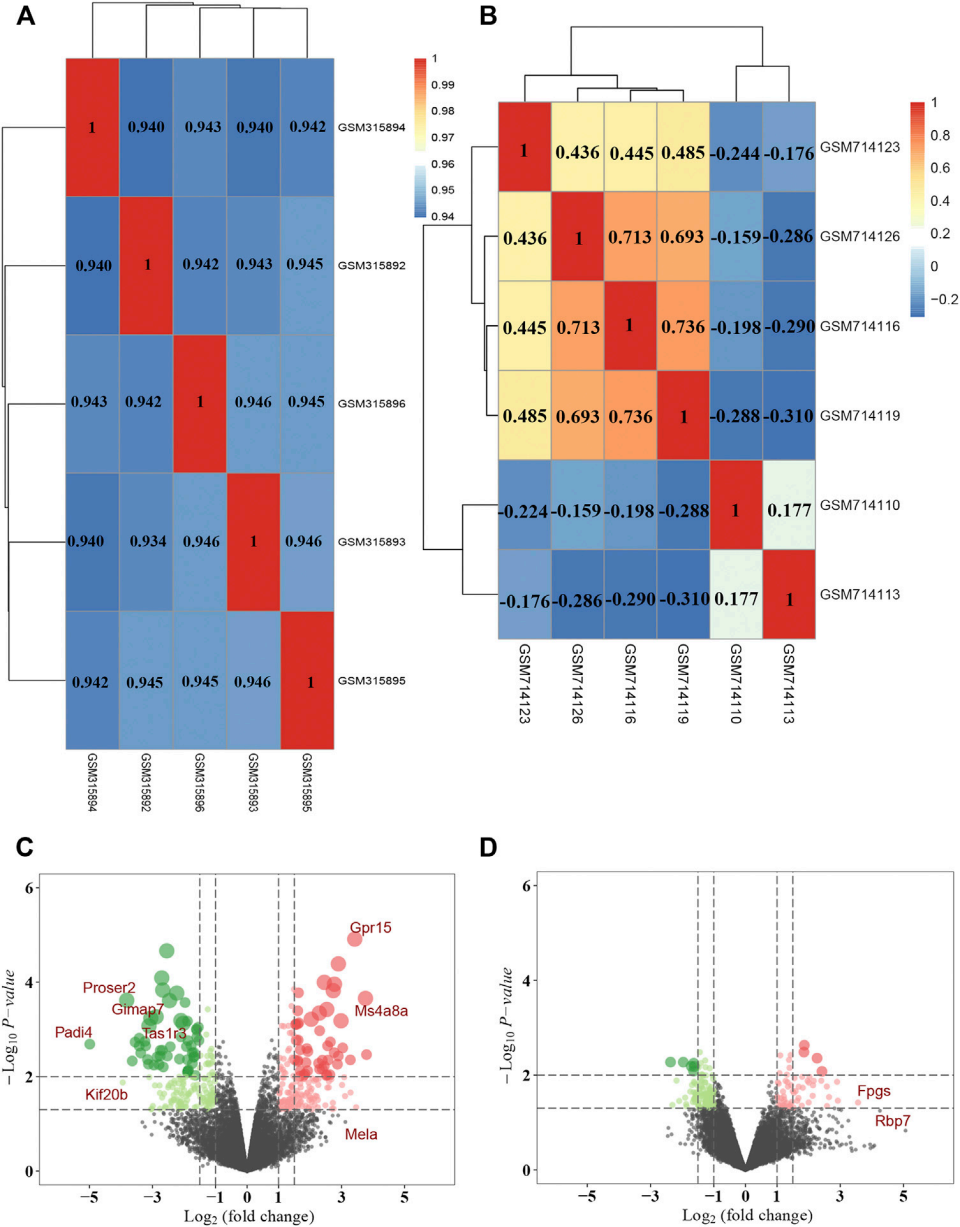
DEGs identification in DR through data mining out of GEO datasets. Pearson correlation analysis of **(A)** GSE12610 dataset and **(B)** GSE28831 dataset. Color reflects the strength of the correlation. The correlation coefficient between 0 and 1 indicates a positive correlation, and a negative correlation between −1 and 0. The greater the absolute value of the number is, the stronger the correlation is. **(C)** The volcano map showed the differential expression between the normal group and the DM group in GSE12610 dataset, with red dots representing up-regulated genes and green dots representing down-regulated genes. **(D)** The volcano map exhibited differential expression between the control and DR groups in the GSE28831 dataset.

A total of 30 potential targets corresponding to AS-IV were identified from GeneCards, 265 corresponding AS-IV targets were identified from PharmMapper, and 108 potential AS-IV targets were predicted by using Swiss Target Prediction. After data deduplication, 398 potential targets were retained (Supplementary Table S1). Pearson correlation test was used to further verify the repeatability of intra-group data in two GEO datasets. Based on Pearson’s correlation test, there was a correlation among the samples of normal group and a strong correlation among the samples of DM group in the GSE12610 dataset (Figure 4A). Moreover, we found that for GSE28831, a correlation existed among the samples of normal group and a strong correlation among the samples of DM group (Figure 4B). Therefore, the Affymetrix microarray dataset GSE12610 and GSE28831 were normalized. 360 DEGs were screened out of GSE12610 dataset using limma packet (corrected *p* < 0.05, logFC>1/logFC <-1), among which 178 down-regulated genes and 182 up-regulated genes were identified (Figure 4C). In addition, 171 DEGs were identified from the GSE28831 dataset, including 111 up-regulated genes and 60 down-regulated genes (Figure 4D).

### Identification and Analysis of Core Targets Between AS-IV and DR

**FIGURE 5:**
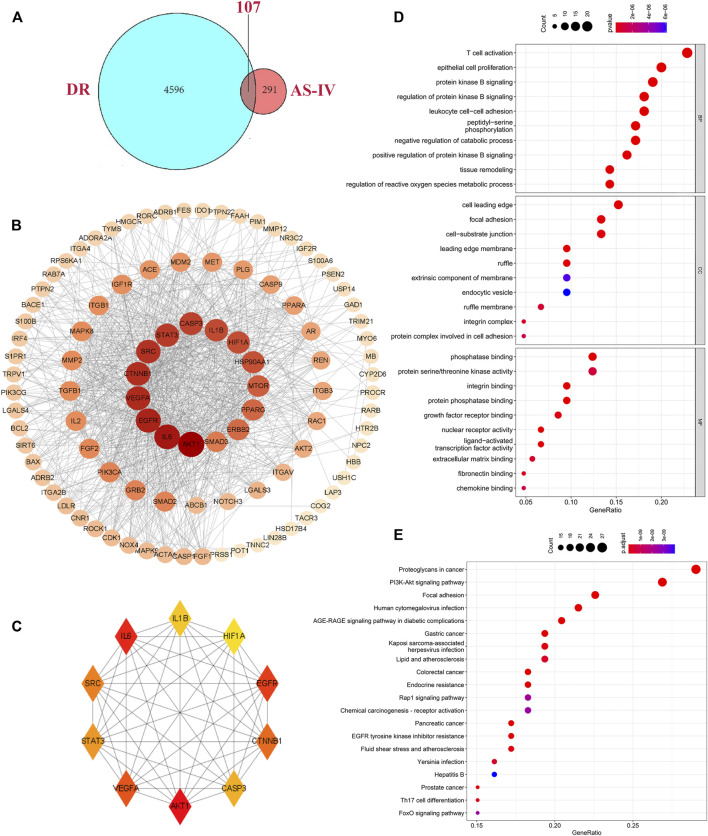
Hub genes identification and biological function analysis. **(A)** Intersection of targets shared by AS-IV and DR. The blue and red ellipses represent the identified DR and AS-IV targets respectively, and the middle part represents the common targets of AS-IV and DR. **(B)** PPI networks were visually analyzed using Cityscape 3.8.2 software. The larger the node area, the darker the color, the more important it is in the network. **(C)** The top 10 key genes obtained by degree value method. Each node represents a protein, and each edge represents the relationship between two proteins. The larger the node in the PPI network the darker the color, the more the node protein will interact with the surrounding proteins. **(D)** GO enrichment analysis of hub genes, including the BP, CC and the MF items. **(E)** KEGG pathways terms involved by hub genes. DR, diabetic retinopathy; AS-IV, astragaloside IV; BP, biological processes; CC, cellular component; MF, molecular function.

A combined analysis of two datasets in GEO database (GSE12610, GSE28831) identified 527 differentially expressed genes associated with DR. In addition, we integrated disease targets from DrugBank, GeneCards, OMIM, PharmGKB, and TTD databases, with 3 genes identified in DrugBank, 4079 genes identified in GeneCards, 85 genes predicted in OMIM, 218 genes in PharmGKB and 27 genes in TTD. Together, 4703 disease targets of DR were determined after integration and de-duplication (Supplementary Table S2). We matched 398 potential targets of AS-IV with 4703 DR target databases, intersecting 107 shared targets (Figure 5A, Supplementary Table S3). The generated PPI network displayed that the network contains 100 nodes with 961 edges (Figure 5B). Hub genes were selected using plugin “cytoHubba” with target connectivity ≥30 (triple the median), and 10 core targets (VEGFA, CASP3, HIF1α, STAT3, CTNNB1, SRC, AKT1, EGFR, IL1β and IL6) out of the overlapping genes with the largest degree value stood out for later analysis (Figure 5C, Supplementary Table S4).

### Biological Functions Analysis of Identified Hub Genes

To explore the biological functions of the core targets, the GO and KEGG pathways were employed. GO analysis results showed a total of 2,269 entries involved by selected targets. In biological processes (BP) enrichment analysis, 2096 items were obtained, of which the top 10 include T cell activation, epithelial cell proliferation, protein kinase B signaling, regulation of protein kinase B signaling, leukocyte cell-cell adhesion, peptidyl-serine phosphorylation, negative regulation of catabolic process, positive regulation of protein kinase B signaling, tissue remodeling, regulation of reactive oxygen species metabolic process (Figure 5D). In the cell component (CC) enrichment analysis, the top 10 out of 68 items include cell leading edge, focal adhesion, cell-substrate junction, leading edge membrane, ruffle, extrinsic component of membrane, endocytic vesicle, ruffle membrane, integrin complex, protein complex involved in cell adhesion (Figure 5D). Molecular function (MF) enrichment analysis revealed 105 items, and the top 10 terms are phosphatase binding, protein Serine/Threonine kinase activity, integrin binding, protein phosphatase binding, growth factor receptor binding, nuclear receptor activity, ligand-activated transcription factor activity, extracellular matrix binding, fibronectin binding, chemokine binding (Figure 5D). KEGG pathway analysis showed that 150 enrichment pathways, of which the most significantly enriched 20 pathways present as proteoglycans in cancer, PI3K-AKT signaling pathway, focal adhesion signaling pathway, human cytomegalovirus infection, AGE-RAGE signaling pathway in diabetic complications, gastric cancer, Kaposi sarcoma-associated herpesvirus infection, lipid and atherosclerosis, colorectal cancer, endocrine resistance, Rap1 signaling pathway, chemical carcinogenesis-receptor activation, pancreatic cancer, EGFR tyrosine kinase inhibitor resistance, fluid shear stress and atherosclerosis, *Yersinia* infection, Hepatitis B, prostate cancer, Th17 cell differentiation, FoxO signaling pathway (Figure 5E, Supplementary Table S5).

### Molecular Docking Analysis of Crucial Targets

**FIGURE 6:**
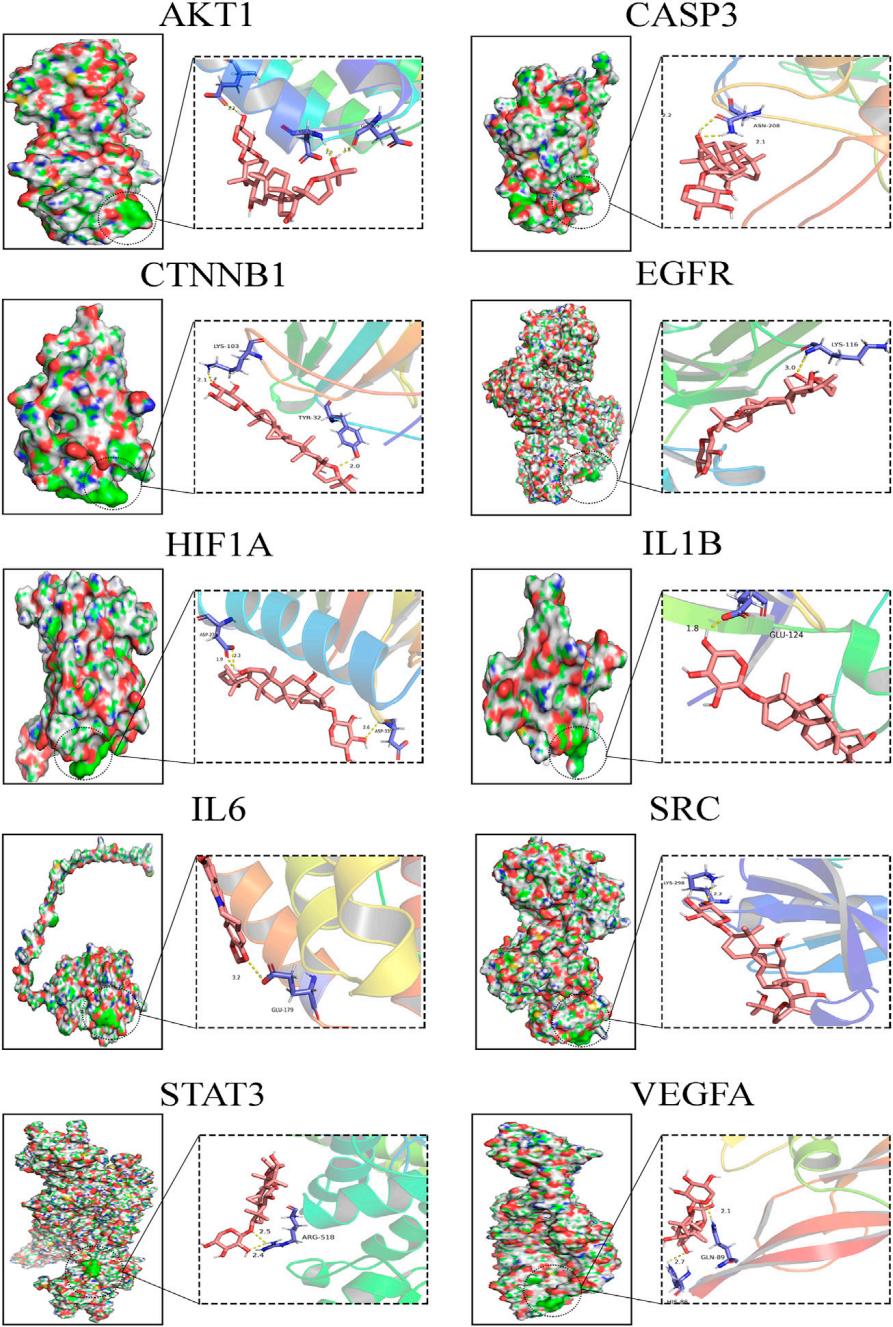
Molecular docking diagrams of AS-IV and hub genes.

In the present study, the possible interactions between 10 hub genes and AS-IV were investigated by molecular docking verification. In the process of molecular model construction, target genes were applied, VEGFA (PDB:1MKG), CASP3(PDB:1QX3), HIF1α (PDB:3HQU), STAT3 (PDB:6TLC), CTNNB1 (PDB:5IVN), SRC (PDB: 4U5J), AKT1 (PDB:5AAR), EGFR (PDB:3G5X), IL1β (PDB: 4X3A), IL6 (PDB:4O9H). Their interaction patterns with key amino acids and their binding at active sites are displayed in Figure 6. The results of binding free energy revealed that the core components of AS-IV have good binding activity with the core targets (Table 2). AS-IV was well connected to the corresponding protein, and the key amino acids around it mainly acted in the form of hydrogen bond. Hydrogen bond formation minimizes the energy of small molecule receptor complexes and is therefore the most stable. The consistent results of molecular docking further demonstrated the reliability of key targets predicated by network pharmacology.

**TABLE 2:** The binding free energy of AS-Ⅳ docking with core target molecules.

Genes	Binding free energy (kcal/mol)	Genes	Binding free energy (kcal/mol)
HIF1A	−4.48	SRC	−2.68
IL6	−3.49	EGFR	−2.31
CTNNB1	−3.16	CASP3	−2.31
IL1B	−3.14	STAT3	−1.41
AKT1	−3.08	VEGFA	−1.08

### Expression Changes of Key Target Genes in Rats with DR After AS-IV Treatment

**FIGURE 7:**
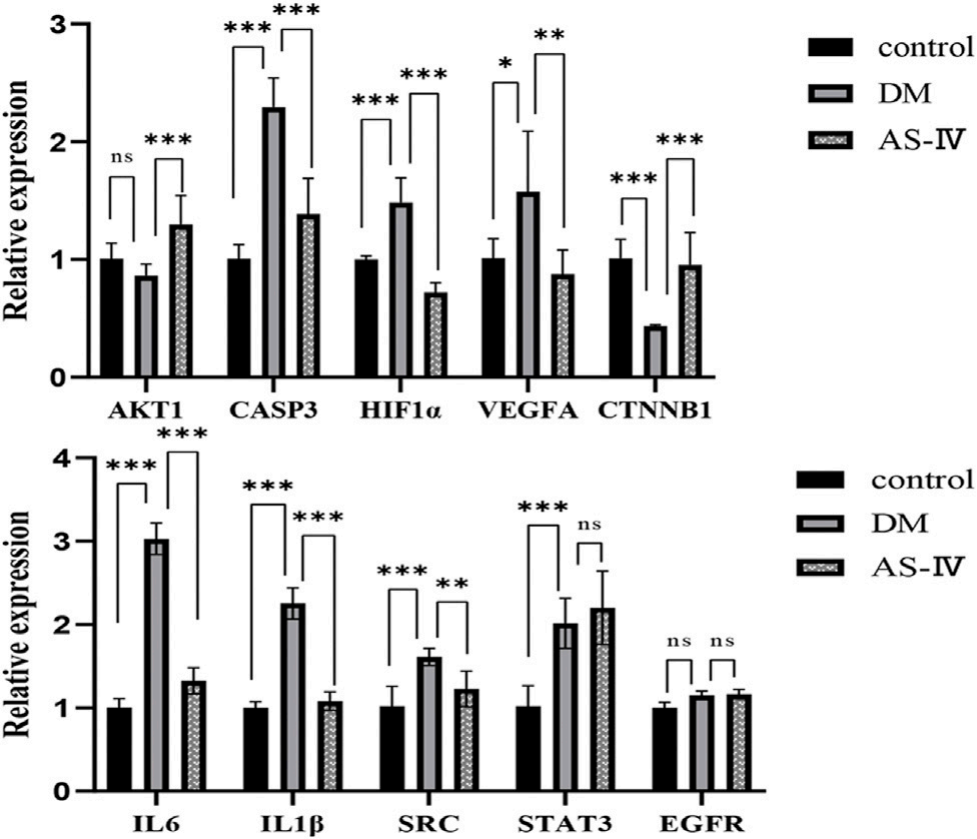
Expression changes of 10 core targets. DM, diabetic group; AS-IV, treatment group. DM, diabetes mellitus; AS-IV, astragaloside IV. Data were presented as mean ± SD, *p < 0.05, **p < 0.01, ***p < 0.001.

RT-qPCR was used to detect mRNA expression changes of core targets identified above in DR rats with AS-IV treatment. Among these genes, levels of CASP3, HIF1α, VEGFA, IL6, IL1β, SRC and STAT3 were significantly elevated after DR insult, which were reversed after AS-IV treatment (Figure 7, *p* < 0.05, *p* < 0.01, *p* < 0.001). On the contrary, AKT1 and CTNNB1 were depressed in diabetic rats, but AS-IV treatment markedly upregulated their expression in DR rats (Figure 7, *p* < 0.001). These results suggest that the expression of AKT1, CASP3, HIF1α, VEGFA, CTNNB1, IL6, IL1β, SRC and STAT3 might play important roles in the treatment of DR with AS-IV.

### AS-IV Treatment Might Enhance Retinal Functions of DR Rats via PI3K-AKT Signaling Pathway

**FIGURE 8:**
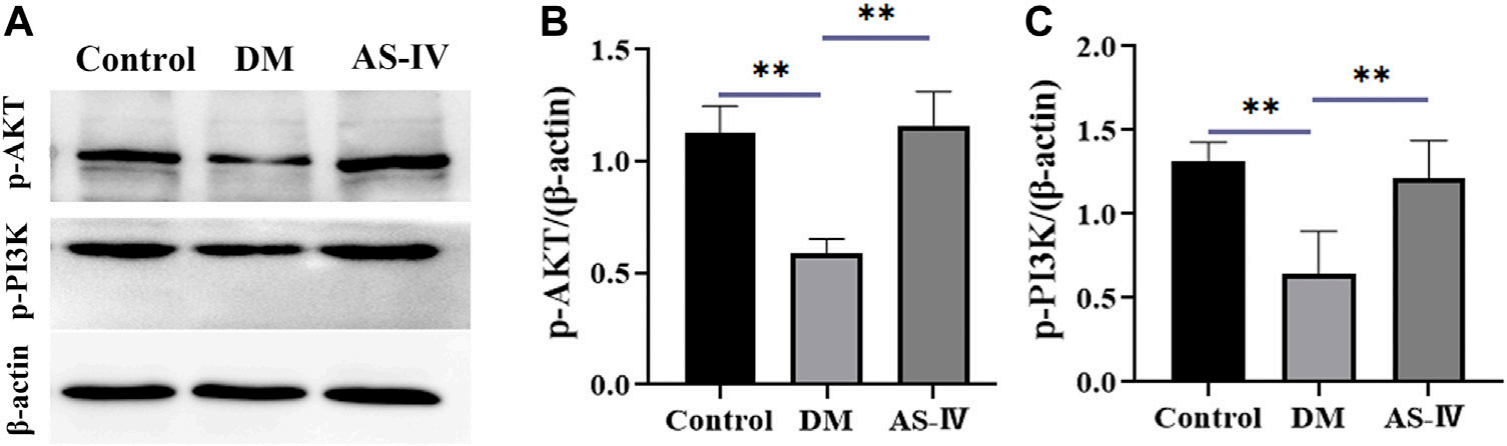
The role of PI3K-AKT signaling in the therapeutic efficacy of AS-IV in DR. **(A)** Protein bands of p-AKT, p-PI3K and β-actin in control, DM and AS-IV groups. Protein levels quantification of **(B)** p-AKT and **(C)** p-PI3K among control, DM and AS-IV groups. DM, diabetes mellitus; AS-IV, astragaloside IV. Data were presented as mean ± SD, **p < 0.01.

AKT is a key downstream signal protein of PI3K, and PI3K/AKT pathway is an important signaling pathway involved in the progression and pathogenesis of DR (Abid, et al., 2004; Cui, et al., 2018). Thus we detected the protein levels of phosphorylated AKT (p-AKT) and p-PI3K to further investigate the therapeutic mechanism of AS-IV in DR. Results of western blot demonstrated that expression of p-AKT and p-PI3K were significantly reduced in DM group compared to that of control group (Figures 8A–C, *p* < 0.01). After AS-IV treatment, their levels exhibited prominent increase relative to that in DM group (Figures 8A–C, *p* < 0.01).

## Discussion

At present, the pathogenesis of DR is complex and remains obscure. This paper mainly combined network pharmacology analysis with experimental verification to explore the molecular mechanism of AS-Ⅳ in the treatment of DR. Our results uncovered that AS-Ⅳ can protect retinal cells against diabetes induced retinopathy, which may act on target proteins AKT1, CASP3 and HIF1α, VEGFA, IL6, IL1β, SRC and CTNNB1, and might be involved in the regulation of PI3K-AKT signaling pathway.

There is increasing evidence that AS-IV (C41H68O14, molecular weight = 784.97) is a bioactive saponin extract of *Astragalus membranaceus* with a wide range of pharmacological effects, including anti-inflammatory and anti-tumor functions (Qu, et al., 2009; Wang, et al., 2019a). A recent study reported multiple protective effects of AS-IV in an animal model of diabetic retinopathy, including reduced apoptosis of retinal ganglion cells, reduced phosphorylation of ERK1/2, inhibition of NF-κB and various cytokines which induce anti-inflammatory effects, and down-regulation of the enzyme aldose reductase (an enzyme involved in the polyol pathway) (Ding, et al., 2014). In another study, AS-IV significantly increased the expression of miR-128 in retinal pigment epithelial cells (RPE), inhibited the apoptosis of RPE cells and alleviated the dysfunction of RPE (Wang, et al., 2020a). AS-IV has also reported to protect retinal capillary endothelial cells from inflammation and oxidative stress induced by high glucose concentrations by lowering GLUT1 and Nox4 expression levels (Qiao, et al., 2017). Consistent in this study, we elucidate the therapeutic efficacy of AS-IV in alleviating retinal damage induced by diabetes revealed by OCT imaging technology and HE staining. All of these highlight the great potentials of AS-IV in treating DR.

To further investigate the underlying mechanism of AS-IV treatment in DR, network-based pharmacology was applied to predict the potential targets of AS-IV and DR in this study. And 107 potential targets of AS-IV for DR were obtained after intersection, among which 10 core targets (VEGFA, CASP3, HIF1α, STAT3, CTNNB1, SRC, AKT1, EGFR, IL1β and IL6) stood out after more precise filtering. RT-qPCR validation of 10 core targets revealed that AKT1, CASP3, HIF1α, VEGFA, IL6, IL1β, SRC and CTNNB1 were modulated in the process of AS-IV treatment in DR, suggesting that AS-IV can be used a potential drug to treat DR through multi targets. It has been previously reported that in proliferative diabetic retinopathy, plasma and vitreous VEGFA levels are high (Han, et al., 2020), and VEGFA permeability is a possible factor in vascular leakage, which has a significant impact on the incidence of diabetic retinopathy (Xie, et al., 2020). VEGFA is not only the most important inducer of vascular leakage, but also a key angiogenic factor (Sharma, 2020). In a previous study, vegfa-flk-1 signaling has been shown to be the main mediator of endothelial cell mitosis, survival and microvascular permeability (Zhang, et al., 2021), suggesting that VEGF can become an important target for the treatment of ocular diseases. Inhibition of HIF-1α in DR can reduce VEGF expression and inhibit neovascularization *in vivo*. VEGF signaling pathway induces activation of PKC and AKT1, promotes endothelial cell proliferation, and increases neovascularization permeability, which is associated with retinal exudation and macular edema in diabetic retinopathy (Wang, et al., 2020b). In addition, HIF-1α is also associated with cell proliferation and inflammation in DR retinas (Min, et al., 2021), and specific knockout of HIF-1α in Muller cells attenuated retinal neovascularization, vascular leakage, and inflammation in streptozotocin induced DR mouse models (Lin, et al., 2011). For microvascular dysfunction of DR, its anti-angiogenesis effect is exerted by inhibiting the expression of VEGF, P-ERK, P-FAK and P-SRC crosstalk (Long, et al., 2019). Reduced mRNA and protein levels of FGF1 and HIF1α inhibit retinal neovascularization in diabetes mellitus (Guan, et al., 2020). Activated SRC is involved in signaling pathways that stimulate endothelial cell survival and angiogenesis (Li, et al., 2018). In hyperglycemic retinal endothelial cells, SRC-SHP2, β-catenin ubiquitination and endothelial permeability were significantly increased (Eshaq and Harris, 2020). In addition, the SRC kinase inhibitor PP2 was found to inhibit HIF1α expression (Jiang, et al., 1997), which suggests that SRC and β-catenin are also closely related to angiogenesis and inflammation.

Mounting evidences indicate that immune mechanism involves in the pathogenesis of DR (Mesquida, et al., 2019). AKT is a regulator of multiple signaling pathways involved in cytotoxic drug-induced inflammation, apoptosis, and autophagy (Yarmohammadi, et al., 2021). It has been reported that activation of PI3K/AKT signal plays an important role in blocking oxidative stress, inhibiting pro-inflammatory response and participating in alleviating the activation of DR microglia (Jacot and Sherris, 2011; Yang, et al., 2017). The PI3K/AKT pathway is also an important signaling pathway regulating cell survival and apoptosis (Franke, et al., 2003). In the PI3K/AKT pathway, phosphatidylinositol-dependent kinase 1 (PDK1) binds to AKT and phosphorylates AKT, thereby activating p70S6K1 and ultimately inhibiting apoptosis (Zhao, et al., 2010; Chai, et al., 2018). Recently, IL-6 has become an important factor in the pathogenesis of DR. The classical IL-6 signaling is associated with anti-inflammatory function, while trans-signaling correlates with pro-inflammatory response (Zegeye, et al., 2018). In a recent study, IL-6^−/−^ mice showed significantly lower levels of superoxide production in the retina in response to angiotensin II stimulation than wild-type mice, suggesting a link between IL-6 and oxidative stress and inflammation in retinal pathology (Robinson, et al., 2020). IL-1 β is known for its role in inducing and amplifying neuroinflammation through its own stimulation (Liu, et al., 2012). Many of the molecular and cellular abnormalities detected in DR support the role of IL-1β in driving neuroinflammation. One separate study demonstrated that IL-1β may function in the development of diabetic retinal glial cell activation and endothelial dysfunction, thus interrupting the vicious cycle triggered by IL-1β auto-stimulation, which may be a mechanism for limiting the progression of DR (Kowluru and Odenbach, 2004).

Given current knowledge of these core targets, it can be inferred that AS-IV protects animals against DR through oxidation resistance, anti-inflammation, anti-apoptosis, inhibition of new blood vessel formation and vascular leakage. Meanwhile, the results of GO enrichment analysis on key targets, showed that the regulation of various kinases activity, insulin response and vascular endothelial cells play a major role in the AS-IV therapy of DR process. The enriched KEGG pathways of these crucial genes mainly include PI3K-AKT signaling pathway, AGE-RAGE signaling pathway in diabetic complications, and EGFR tyrosine kinase inhibitor resistance, suggesting that AS-IV may play an anti-inflammatory role by activating PI3K/AKT signaling pathway. Oxidative stress and inflammation may play an important role in delaying the progression of DR (Cecilia, et al., 2019). PI3K/AKT pathway is an important signaling pathway involved in the progression and pathogenesis of DR, endothelial cell dysfunction, and regulation of VEGF level and inflammation (Abid et al., 2004; Cui et al., 2018). AKT is a key downstream signaling protein of PI3K, and activated PI3K binds to AKT, which promotes the expression and secretion of pro-inflammatory cytokines through the activation of NF-κB pathway, resulting in imbalance of cytokine secretion and a series of inflammatory reactions (Dhandapani, et al., 2005; Koorella, et al., 2014; Shi, et al., 2016). A number of studies have shown that inhibition of PI3K signal can inhibit the secretion of pro-inflammatory factors and increase the secretion of anti-inflammatory factor IL-10 when toll-like receptor- (TLR-) inflammation occurs (Fallah, et al., 2011; Bai, et al., 2014). It has also been reported that blocking the PI3K/Akt/mTOR signaling pathway can reduce cell viability by destroying the functions of Akt and RhoB, which is conducive to the treatment of DR (Zhang, et al., 2019b). A study reported that PI3K is induced by VEGF/VEGFR2, which enhances PI3K activation through phosphorylation, and VEGF can activate AKT pathway in a PI3K-dependent manner (Wang, et al., 2019b). In fundamental agreement with the previous finding, we conducted western blot to detect the protein levels changes of AKT and PI3K, and the results revealed that AS-IV treatment reversed the DR-induced inhibition on levels of AKT and PI3K, implying AS-IV might play protective efficacy through activating PI3K-AKT signaling.

## Conclusion

Collectively, this study identified core targets underlying the therapeutic effects of AS-IV in DR based on comprehensive network pharmacology, and preliminarily unveiled the therapeutic effects of AS-IV might be achieved via activation PI3K/AKT pathway, providing a strong theoretical and experimental basis for the follow-up clinical application of AS-IV in the treatment of DR.

## Data Availability

The datasets presented in this study can be found in online repositories. The names of the repository/repositories and accession number(s) can be found in the article/Supplementary Material.
